# Ultrastructural Localization and Molecular Associations of HCV Capsid Protein in Jurkat T Cells

**DOI:** 10.3389/fmicb.2017.02595

**Published:** 2018-01-04

**Authors:** Cecilia Fernández-Ponce, Maria C. Durán-Ruiz, Isaac Narbona-Sánchez, Juan P. Muñoz-Miranda, Mikel M. Arbulo-Echevarria, Antonio Serna-Sanz, Christian Baumann, Rocío Litrán, Enrique Aguado, Wilhelm Bloch, Francisco García-Cozar

**Affiliations:** ^1^Department of Biomedicine, Biotechnology and Public Health, University of Cadiz and Institute of Biomedical Research Cádiz (INIBICA), Cadiz, Spain; ^2^Sciex Darmstadt, Darmstadt, Germany; ^3^Department of Condensed Matter Physics, University of Cádiz, Puerto Real, Spain; ^4^Department of Molecular and Cellular Sport Medicine, Institute of Cardiovascular Research and Sport Medicine, German Sport University Cologne, Cologne, Germany

**Keywords:** Hepatitis C virus, immune evasion, proteomics, interactome, ultrastructure, regulatory T cells, immune tolerance

## Abstract

Hepatitis C virus core protein is a highly basic viral protein that multimerizes with itself to form the viral capsid. When expressed in CD4^+^ T lymphocytes, it can induce modifications in several essential cellular and biological networks. To shed light on the mechanisms underlying the alterations caused by the viral protein, we have analyzed HCV-core subcellular localization and its associations with host proteins in Jurkat T cells. In order to investigate the intracellular localization of Hepatitis C virus core protein, we have used a lentiviral system to transduce Jurkat T cells and subsequently localize the protein using immunoelectron microscopy techniques. We found that in Jurkat T cells, Hepatitis C virus core protein mostly localizes in the nucleus and specifically in the nucleolus. In addition, we performed pull-down assays combined with Mass Spectrometry Analysis, to identify proteins that associate with Hepatitis C virus core in Jurkat T cells. We found proteins such as NOLC1, PP1γ, ILF3, and C1QBP implicated in localization and/or traffic to the nucleolus. HCV-core associated proteins are implicated in RNA processing and RNA virus infection as well as in functions previously shown to be altered in Hepatitis C virus core expressing CD4^+^ T cells, such as cell cycle delay, decreased proliferation, and induction of a regulatory phenotype. Thus, in the current work, we show the ultrastructural localization of Hepatitis C virus core and the first profile of HCV core associated proteins in T cells, and we discuss the functions and interconnections of these proteins in molecular networks where relevant biological modifications have been described upon the expression of Hepatitis C virus core protein. Thereby, the current work constitutes a necessary step toward understanding the mechanisms underlying HCV core mediated alterations that had been described in relevant biological processes in CD4^+^ T cells.

## Introduction

Hepatitis C virus (HCV) infection is an important cause for chronic viral liver disease and one of the main indications for liver transplantation (Anzola, 2004; Dustin and Rice, 2007). HCV affects 80 million people worldwide and in more than 80% of the patients leads to chronicity (Gower et al., 2014). The high level of chronicity and the absence of a protective vaccine, makes HCV infection a significant public health problem (Anzola, 2004; Dustin and Rice, 2007; Gower et al., 2014).

The molecular mechanisms harnessed by HCV to establish a chronic infection and their implications in the innate and adaptive immune systems have not been fully elucidated. In this regard, several HCV viral proteins have been described as modulators of immunological phenomena (Yao et al., 2007; Krishnadas et al., 2010; Tu et al., 2012; Chen et al., 2015). Among them, HCV core protein has been widely associated with pathogenicity, virulence, immune evasion and immune regulation (Dominguez-Villar et al., 2007, 2012a,b; Waggoner et al., 2007; Tu et al., 2012; Doumba et al., 2013; Fernandez-Ponce et al., 2014, 2017). However, the underlying molecular processes, as well as the behavior of HCV core protein or its interactions with host cell components, remain unclear.

HCV core is a highly basic protein, which binds and protects the viral RNA, multimerizing with itself to form the viral capsid (Santolini et al., 1994). In mammalian infected cells, HCV core interacts with endoplasmic reticulum membranes, lipid droplets and other cellular and viral proteins to promote assembly of new virions. However, several inhibitory molecules and the lack of some host factors can hamper viral production by disrupting the core multimerization process and the coordinated interactions with other viral proteins (Mousseau et al., 2011; Gawlik and Gallay, 2014). In this way, non-enveloped HCV core proteins can be directed to alternative subcellular locations and also be released into the extracellular space (Maillard et al., 2001; Polyak et al., 2006; Tan et al., 2006).

According to these findings, in HCV chronically infected patients, HCV core protein, has been detected as non-enveloped isolated nucleocapsids, not only in hepatocytes (Falcon et al., 2003), but also in serum (Maillard et al., 2001), peripheral blood CD4^+^ T cells (Fernandez-Ponce et al., 2014) and other non-parenchymal liver cells, such as, lymphocyte, pit, endothelial, stellate, Kupffer and polymorphonuclear cells (Falcon et al., 2005). In T cell lines, non-enveloped isolated nucleocapsids binding and internalization has also been described *in vitro* (Doumba et al., 2013). These data further support the presence of HCV core protein inside immune cells, including lymphocytes, during HCV chronic infection.

Interestingly, HCV core protein intracellular expression in CD4^+^ T lymphocytes has been shown to induce modifications in cell proliferation, cell cycle progression, expression of anergy genes, transcription of genes involved in cytoskeleton reorganization, vesicle trafficking, endocytosis, transcription and translation, cytokine production, cell death and generation of a T cell regulatory phenotype with exhausted features (Bergqvist and Rice, 2001; Bergqvist et al., 2003; Dominguez-Villar et al., 2007, 2012a; Doumba et al., 2013; Fernandez-Ponce et al., 2014), characterized by an increased expression of Foxp3 (forkhead box P3) and CTLA-4 (cytotoxic T-lymphocyte antigen-4) (Dominguez-Villar et al., 2012a), high levels of IL-10 secretion, and decreased IL-2 and IFN-γ production (Doumba et al., 2013; Fernandez-Ponce et al., 2014).

It has been described for several viruses that the specific subcellular localization of viral proteins and their interactions with host molecules can alter the spatial distribution and organization of cellular proteins, and in this way, induce diverse molecular and cellular effects (Chen et al., 2002; Yoo et al., 2003; Ning and Shih, 2004; Bertrand and Pearson, 2008; Ponti et al., 2008; Hiscox et al., 2010; Zhu et al., 2013; Raval et al., 2015).

As studies using the whole virus do not allow for the elucidation of the specific molecular mechanisms in which each protein is implicated, in this work, we focused on a single viral protein, showing that in CD4^+^ T cells, HCV core protein mostly localizes in the nucleus and specifically in the nucleolus where it is greatly enriched. In addition, we performed pull down assays, combined with Mass Spectrometry analysis, in order to identify host proteins associated with HCV Core, which could be implicated in the functional effect previously observed to be induced by the presence of HCV-core in T cells. We found several proteins implicated in important functions that are associated with HCV core protein. Thereby, our results shed light on the molecular mechanisms underlying the alterations in biological cell processes and the generation of adaptive regulatory-like CD4^+^ T cells in the periphery by the intracellular presence of a single HCV viral protein.

## Materials and methods

### Cell cultures

Human Embryonic Kidney (HEK) Lenti-X™ 293T cell line (Clontech) and Jurkat cell line (American Type Culture Collection, Manassas, VA, USA) were maintained in Dulbecco's modified Eagle's medium (DMEM™) supplemented with 10% (v/v) heat inactivated Fetal Bovine Serum (FBS), 2 mM L-glutamine, 10 mM Hepes, 1% (v/v) sodium pyruvate, 50 μM 2-mercaptoethanol, 100 U/ml penicillin and 100 μg/ml streptomycin at 37°C, 10% CO_2_. Human peripheral blood samples were obtained from healthy donors upon signature of an informed consent and following approval by the Ethic sub-commission of the Puerta del Mar University Hospital (dependent from the Central Quality Commission), in accordance to Spanish and European Union Regulations. Peripheral Blood Mononuclear Cells (PBMCs) were isolated by density gradient centrifugation using Lymphocyte separation medium (EurobioTM, Montpellier, France). Cells were washed three times with PBS, subsequently stimulated with 1 mg/ml phytohemagglutinin-P (PHA) (SigmaTM, Saint Louis, Missouri, USA) and cultured in DMEM supplemented with 1% (v/v) sodium pyruvate, non-essential aminoacids, vitamins, L-arginin, L-asparragin, folic acid, 10 mM Hepes, 50 mM 2-mercaptoethanol, 100 mg/ml streptomycin, 100 U/ml penicillin (Life Technologies, Carlsbad, CA, USA) and 10% heat-inactivated FBS (Gibco) at 37°C, in a 10% CO2 atmosphere. 40 U/ml IL-2 was added to the cultures every 48 h, for a total of 5 days to obtain blasts.

### Lentiviral production

HEK Lenti-X™ 293T cells (Clontech) were used as packaging cell lines to produce lentiviral supernatant by co-transfecting plasmids pCMVDR8.91, coding for HIV-1 GAG and POL proteins and pMD2.G for the Vesicular Stomatitis Virus G protein (VSVG) together with the transfer vector pHRSincPPT-SEWHCV-core-GFP (HCV-core) coding for the first 191 amino acids of the HCV polyprotein (serotype 1a) corresponding to HCV-core protein. Transfer vector pHRSincPPT-SEWGFP expressing only GFP (Green fluorescent protein) was used as a control (Dominguez-Villar et al., 2007). Cells were transfected in OptiMEM™ medium using 10 cms diameter cell-culture dishes coated with Collagen (Collagen I, Rat Tail Gibco® Invitrogen cell culture), and Polyethylenimine (PEI), branched (Sigma-Aldrich) as cationic polymer. Transfection efficiency was evaluated by FACS analysis. Supernatants were collected at 48 and 72 h, centrifuged to remove cells and debris and concentrated using Lenti-X™ Concentrator (Clontech®) to obtain a high-titer virus-containing pellet. Pellets were frozen at −80°C and stored until use. Viral titer in supernatants was determined evaluating their efficiency in infecting Jurkat cells.

### Jurkat cell transduction

Jurkat cells were transduced using HCVcore-GFP and GFP (as control) lentiviral supernatants, previously prepared, at a multiplicity of infection (MOI) of 10. Aliquots from the cultures were collected after 48 h to determine the transduction efficiency. GFP expression was monitored by FACS (CyanADP-MLE™; DakoCytomation™) presenting a transduction range >90% in all experiments.

### HCV core protein localization analysis in jurkat cells

#### Transmission electron microscopy

Untransduced, HCVcore-GFP and GFP transduced Jurkat cells were collected 48 h post-transduction, counted, washed with phosphate buffer saline (PBS) and fixed for 24 h using 4% paraformaldehyde (PFA) in PBS, pH 7.37. Subsequently, cell pellets were pre-embedded in agarose 1x, taken out of the tube with a needle and sliced into approximately 1 mm^3^ pieces.

#### Immunoelectron microscopy

Pre-embedding Immunogold was performed. Agarose pieces were washed in PBS, permeabilized using 0.05% Triton X−100 in PBS and incubated in Aurion blocking solution containing normal goat serum (Aurion® 905.002), for 60 min at 4°C. Sections were stained immunochemically with anti-GFP antibody (Abcam ab6556) diluted 1:1,000 in PBS containing 0.2% bovine serum albumin (BSA) at 4°C, overnight, and subsequently washed with the same buffer. For gold particle staining, sections were incubated with 10 nm gold-labeled anti-rabbit IgG (Sigma-Aldrich) diluted 1:50 in 0.2% BSA/PBS at 4°C, overnight. Agarose pieces were washed in 0.1 M Caco-Buffer pH 7.37 at room temperature.

Sections were post-fixed in 0.5% osmium tetroxide and 0.1 M Caco-buffer at room temperature, for 3 h and washed with 0.1 M Caco-buffer. Fixed sections were dehydrated by sequential incubation in ethanol 50–100% and propylene oxide, embedded in epoxy resin and polymerized at 62°C for 72 h. Semi (500 nm) and ultra-thin (50–70 nm) sections, were obtained using a Leica EM UC7 ultramicrotome. Uultra-thin sections were collected onto plastic-coated nickel grids and immunochemically contrast-stained with uranium salts (uranyl acetate) and lead citrate to reveal cell ultrastructure. Finally, samples were analyzed using a transmission electron microscope EM902A Fa.Zeiss with the iTEM Soft Imaging System software (Olympus).

### HCV core protein pull-down assay

#### Cell lysis and protein extraction

Untransduced Jurkat cells and PBMCs cultured as was previously described, were collected, counted, washed with PBS and 10^6^ cells were incubated 30 min with 100 μl lysis buffer (50 mM Tris-HCl pH 7.4, 150 mM NaCl, 1% NonidetP-40, 1 mM EDTA, 1 mM phenylmethylsulfonyl fluoride, 1 mM Na_3_VO_4_, 1 mM NaF, on ice. Cell debris was discarded by centrifugation at 12,000 g for 20 min, 4°C, and soluble proteins were stored at −80°C before further analysis. Protein levels were measured using Bradford protein assay kit (Pierce), according to manufactures recommendations.

#### Pull-down assay

Two hundred microliter magnetic beads (Dynabeads® MyOne™ Streptavidin T1 Invitrogen) were washed following manufacturer's recommendations and conjugated with HCV core Genotype-1b Biotin-ProSpec (HCV-242) by incubating 100 μl (10 mg/ml) Dynabeads with 100 μl (800 pmoles) HCV core protein in PBS, for 2 h at 4°C, with gentle rotation. HCV core protein-coated beads were washed three times by resuspension in PBS containing 0.02% Twin-20 and decanting the supernatant while placed on a magnet. For Mass Spectrometry analysis, coated dynabeads (or uncoated as control for unspecific binding) were incubated overnight, at 4°C, with Jurkat cells protein lysates, with gentle rotation, in the following ratio: 100 μl of coated beads with 150 μg of total protein lysate. For Western blot experiments, coated dynabeads were incubated overnight, at 4°C, with protein lysates from PBMC blasts, with gentle rotation, in the following ratio: 50 μl coated beads with 3 mg protein sample. 50 μl uncoated beads were incubated with 3 mg protein lysate, in the same conditions, as a control of unspecific binding.

#### Mass spectrometry analysis and protein identification

Samples were washed twice with PBS and once with ultrapure water and incubated with 150 μl of 200 mM dithiothreitol (DTT) in 50 mM ammonium bicarbonate for 5 min at 65°C. Eluated proteins were quantified and 100 μg were precipitated with acetone during 1 h at −20°C and centrifuged 30 min at 12,000 g. Subsequently, proteins were resuspended in urea 6 M, reduced with 2 mM DTT in 100 mM ammonium bicarbonate at room temperature for 45 min and alquilated with 4 mM Iodacetamide 45 min, in the dark, subsequently trypsin digestion was performed with sequencing grade trypsin (Promega, Madison, WI, USA) at 37°C overnight (enzyme/substrate ratio 1:50). Finally, the digestion was quenched adding 5% formic acid. The resulting peptides were transferred to a clean tube, dried in speed-Vac and stored at −20°C prior to analysis by mass spectrometry (MS).

Peptides were resolved by reverse phase chromatography using an Eksigent Ultra pump fitted with a 75 μm ID column 25 cm length (Acclaim PepMap 100 C18 2 μm 100 A). Samples were initially loaded for desalting into a 2 cm length 100 μm ID precolumn packed with the same chemistry as the resolving column prior to analytical chromatography. Mobile phases used were 100% Water 0.1% formic acid (buffer A), 100% Acetonitrile 0.1% formic acid (buffer B). Gradient was developed during 95 min up to 35% B. Column was equilibrated in 95% B for 10 min and 5% B for 15 min, using a constant flow of 300 nl/min. Peptides eluted from the column were analyzed using a Sciex 5600 TripleTOF™ system. Data dependent acquisition was collected upon a survey scan performed in a mass range from 350 to 1,250 m/z in a scan time of 250 ms. The top 25 most intense precursors were selected for fragmentation with a transmission window width of 0.7 Da. Minimum accumulation time for MS/MS was set to 75 ms giving a total cycle time of 2,125 ms. Fragment ions were collected in a mass range from 230 to 1,500 m/z in order to have a single Q2 transmission window. Precursors were excluded from further fragmentation during 15 s.

Raw data files were processed using ProteinPilot™ 4.5 software from Sciex and mgf files were loaded onto ProteinScape™ 3.1 software (Bruker) for data grouping and protein identification via Mascot program version 2.5 (Matrix Science Ltd, London, UK). Search parameters were: Homo sapiens taxonomy. Trypsin specificity was used and only one missed cleavage allowed, cysteine carbamidomethylation as fixed and oxidized methionine was used as variable modification. Mass tolerance of precursor ion and fragment ions were 10 ppm and 0.05 Da, respectively. Peptides charges of 1+, 2+, and 3+ were selected and minimum peptide length was set at five residues. Peptides and proteins were considered positively identified if their Mascot score was higher than 20 and 30, respectively. Proteins were identified with the minimum of one peptide and a false discovery rate <1%. Proteins identified with one single peptide were only included if peptide scores were significant, and peptide spectra contained most “y” and/or “b” representative ions for the corresponding peptide sequence. Examples are included in Supplementary Table 2.

Subsequently, we compared the identified proteins that bound to the dynabeads vs. the ones identified binding to HCV core protein using the “Compare Results” option from ProteinScape software 3.1. Thus, 222 proteins were found to specifically associate with HCV core protein.

Proteome profiling data has been deposited in the PeptideAtlas repository, identified as PASS00982.

### Electrophoresis and western blot analysis

Samples obtained from the pull down assay were washed twice with PBS using the magnet, and incubated with 2X Laemmli buffer at 99°C for 5 min. Subsequently, beads were removed from the proteins using a magnet and proteins resolved by SDS-PAGE and transferred to PVDF membranes (Immobilon-FL). Membranes were incubated with the corresponding primary antibody, followed by the appropriated secondary antibody, conjugated to HRP. Primary antibodies used were PP1 gamma antibody (PA5-21671) from Thermo Fisher Scientific® and MYPT1 antibody (#2634) from Cell Signaling Technology®. Reactive proteins were visualized using an ECL system and images were acquired in a ChemiDoc Touch Imaging System (Bio-Rad® Laboratories, Hercules, CA, USA).

#### Localization and network analysis of identified candidate proteins associated with HCV core protein

STRING database version 10 (Search Tool for the Retrieval of Interacting Genes/Proteins) (http://string-db.org) (Szklarczyk et al., 2015) was applied to visualize detailed information about the subcellular localization and the main interactions of the identified proteins. Ingenuity Pathways Analysis (IPA; Ingenuity Systems, Redwood City, Calif.) software was used for further protein characterization according to known and predicted associations into function canonical pathways and networks recorded in the IPA library.

### Prediction of nucleolar localization sequences (NolSs) in HCV core protein using a bioinformatics web server (NoD)

Nuclear localization sequence (NolSs) in HCV core protein prediction was obtained by entering a protein sequence in Fasta format on the NoD web server http://www.compbio.dundee.ac.uk/www-nod/ (Scott et al., 2011). NoLSs were predicted if the average score output by the artificial neural network ANN of 8 consecutive windows was at least 0.8 (Scott et al., 2010, 2011).

## Results

### HCV core protein localizes in the nucleus with enrichment in the nucleoli in jurkat cells

**Figure 1:**
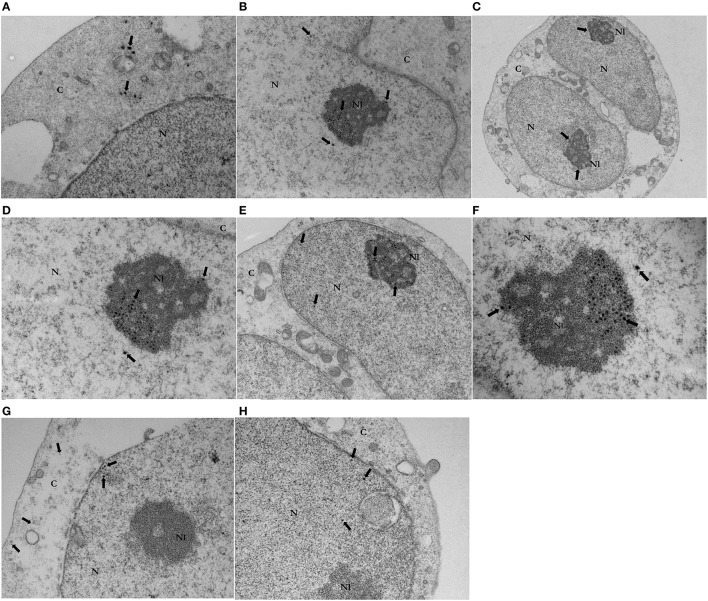
Transmission Electron Microscopy of HCV core-GFP **(A–F)** or GFP **(G,H)** transduced Jurkat cells. Images **(A)** (x20000), **(B)** (x12000), and **(C)** (x7000) are representative micrographs from GFP HCV core - expressing Jurkat cells. Gold particles indicate the position of immunolabeled HCV core-GFP inside Jurkat cells. Immunostaining (arrows) revealed GFP HCV core protein in the nucleus [N] **(B)**, cytoplasm [C] **(A)** and mainly in the nucleoli [Nl] **(B,C)**. Micrographs **(D)** (x20000), **(E)** (x12000), and **(F)** (x30000) show a magnification of the gold labeled HCV core-GFP expressing Jurkat cell. This closer inspection confirmed gold particles labeling GFP core protein (arrows) in the nucleus, with enrichment and a clustering distribution in the nucleoli. Images **(G)** (x20000) and **(H)** (x20000) are representative micrographs of GFP-expressing control Jurkat cells. Gold particles indicate the position of immunolabeled GFP. Immunostaining (arrows) revealed GFP in the cytoplasm **(G)** and nucleus **(G,H)**. No immunostaining was observed in the nucleoli in GFP control cells **(G,H)**.

To analyze the localization of HCV core protein in T cells and to circumvent the lack of optimal anti-HCV core antibodies, we took advantage of a GFP-HCV core fusion construct that has been extensively used in our laboratory (Dominguez-Villar et al., 2007, 2012a,b; Fernandez-Ponce et al., 2014) and an unfused GFP expressing construct as a control. Jurkat cells were efficiently transduced with lentiviral vectors expressing HCVcore-GFP or GFP as a control. Percentage of transduced cells analyzed by flow cytometry was >98% in all cases (data not shown). Jurkat cells transduced with GFP or HCV core GFP- expressing lentiviral construct or left untransduced were subsequently immunostained with anti-GFP antibody and a 10 nm gold-labeled anti-rabbit IgG and analyzed by transmission electron microscopy. As shown in Figure 1, HCV core was mostly localized in the nucleus (Figures 1B,D,E) and specifically in the nucleolus where it was greatly enriched (Figures 1B–F), although some immunostaining was observed in cytoplasm (Figure 1A). HCV-core-GFP was detected in the nucleus in 35% out of 40 HCV Core GFP expressing Jurkat cells analyzed, 22% showed the presence of GFP inside both nucleus and cytoplasm, 5% of the cells only in cytoplasm (Figure 1A), while 18% showed GFP immunolabelling in nucleus with enrichment in the nucleolus (Figures 1B–F). 20% of the cells were not stained. In HCV Core expressing Jurkat cells which showed Core protein nucleolar localization, the total raw number of gold particles counted inside the nucleoli was 196, with an average of 28 gold particles per cell and a Standard Deviation of 13.3.

Regarding GFP transduced control cells, GFP was localized in the nucleus of 84% from 40 GFP-expressing Jurkat cells analyzed and in both nucleus and cytoplasm in 16%. There was no recognizable co-localizing with any organelle. Nucleolus localization was not visible in any cell (Figures 1G,H). No immunogold staining was observed in untransduced Jurkat cells (data not shown). Thus, the study revealed specific immunolabeling of HCV core protein in the nucleolus of Jurkat cells.

### Proteomic analysis of HCV core associated-proteins. subcellular localization and protein function

**Figure 2:**
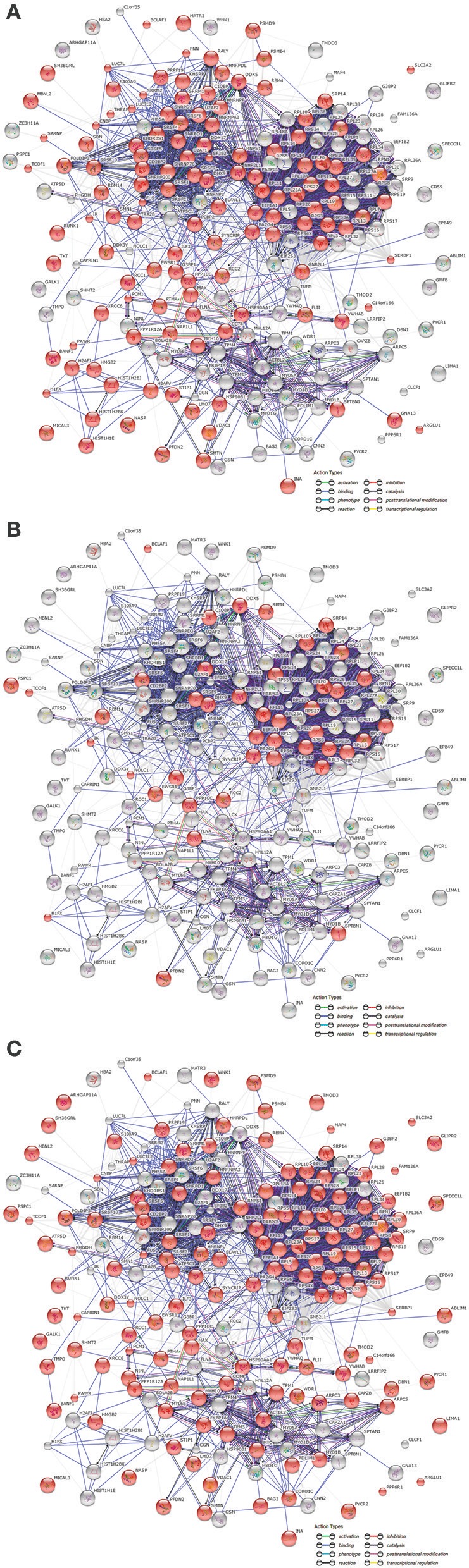
Subcellular localization of proteins associated with HCV core protein. Associated proteins were classified by String™ 10 software using the “actions” view, according to the “GO cellular components” distribution option, in order to identify their subcellular localization. String analysis reported that proteins were nuclear **(A)** (128 from 222), nucleolar **(B)** (43 from 222), and cytoplasmic **(C)** (150 from 222). Colored lines between proteins indicate the type of evidence for each interaction, with a minimum required confidence score of 0.4.

Based on the findings obtained with the electron microscopy assay, we decided to identify HCV core associated proteins that could mechanistically be correlated with HCV core protein nucleolar localization. Thus, we performed pull down experiments using biotinilated HCV core protein as bait. Further analysis with the String platform allowed us to classify the identified proteins depending on their localization (Figure 2). Thus, from the 222 associated proteins identified (Supplementary Table 1), 128 have been previously described to localize in the nucleus (Figure 2A) and 43 in the nucleolus (Figure 2B), while most of the 150 proteins described in the cytoplasm (Figure 2C) have also been identified in the nucleus and/or nucleolus. String also allowed us to determine potential protein functionalities according to previously published reports. Interestingly, most of the identified HCV core associated proteins were found to participate in binding processes, as indicated in Figure 2.

**Figure 3:**
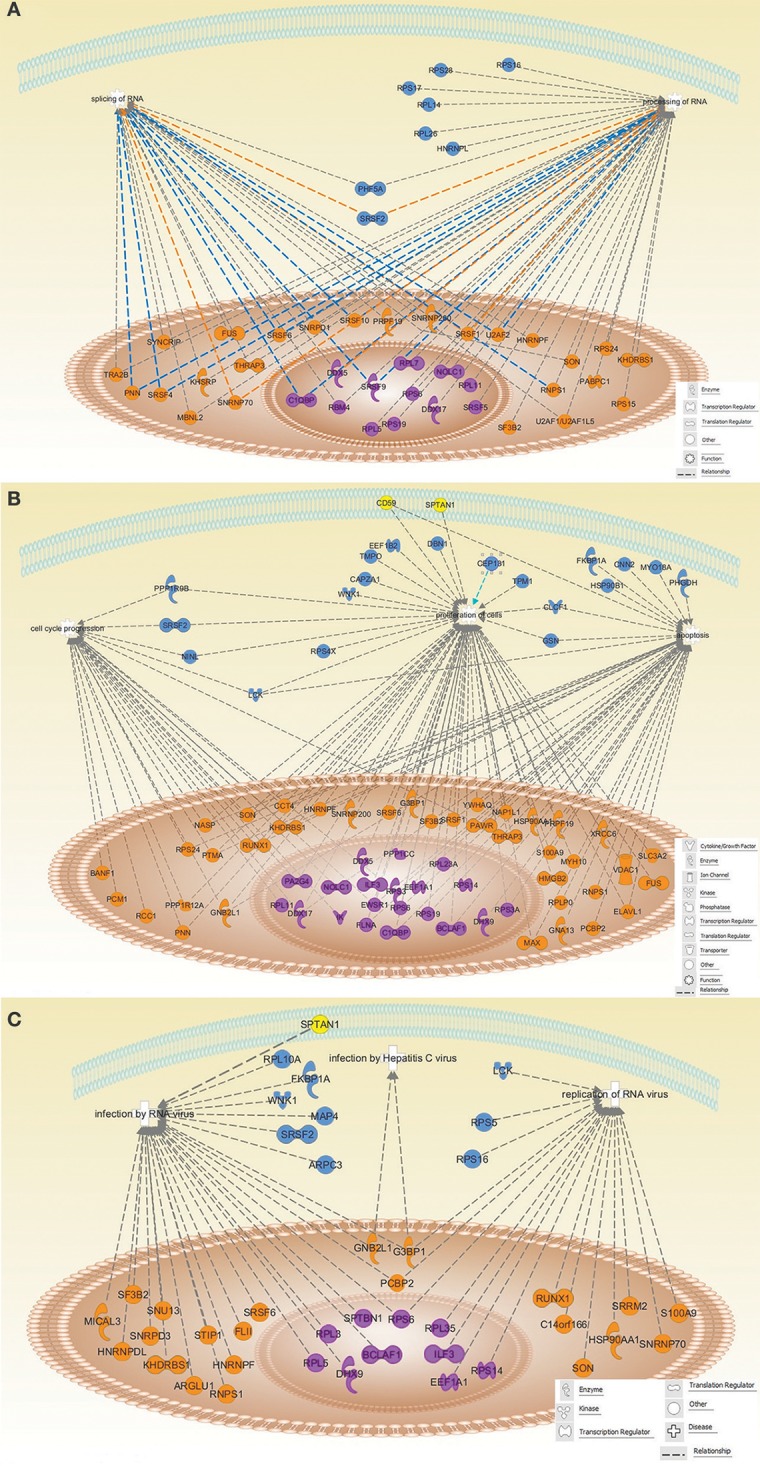
Schematic representation of the identified proteins involved in different top networks categories. The identified proteins were associated with splicing and processing of RNA **(A)**; cell cycle progression, proliferation and apoptosis **(B)**; infection by RNA viruses, replication of RNA viruses and Hepatitis C virus infection **(C)**. Protein shapes indicate their function. Proteins are localized in the nucleus (orange), nucleolus (violet), cytoplasm (blue), and plasma membrane (yellow), according to String 10 Go Cellular component. All the relationship lines are supported by at least one reference from the literature, textbooks or canonical information stored in the Ingenuity Knowledge Base.

Regarding protein function, we mainly focused on the functional classification given by the IPA software, which, based on biomedical literature and integrated databases, allows to determine the most probable pathways and/or functions in which identified proteins are involved. Thus, IPA core analysis of our dataset (Figure 3) revealed that the identified HCV core associated proteins were mainly described to participate in splicing and processing of RNA, cell cycle progression, cell proliferation, apoptosis and RNA virus infection. Our findings support the concept that the subcellular localization of HCV core protein might have an impact on CD4^+^ T cells functions.

### Western blot validation of mass spectrometry analysis

In order to confirm, by an alternative method, some of the associations identified by Mass spectrometry analysis, and most importantly to evaluate whether such associations were also present in primary T cells (PBMC), thus enhancing the relevance of our findings. Postnuclear lysates from human PBMCs blasts (see methods), were subjected to HCV Core protein pull down, using a biotinilated HCV Core protein (800pmoles) bound to magnetic beads as a bait. Uncoated magnetic beads were used as a control. Experiments were first carried out in Jurkat cells and inputs from Jurkat and PBMC were loaded in parallel (1.5% from the amount used per pull down), to evaluate the relative amount of each target protein (data not shown).

**Figure 4:**
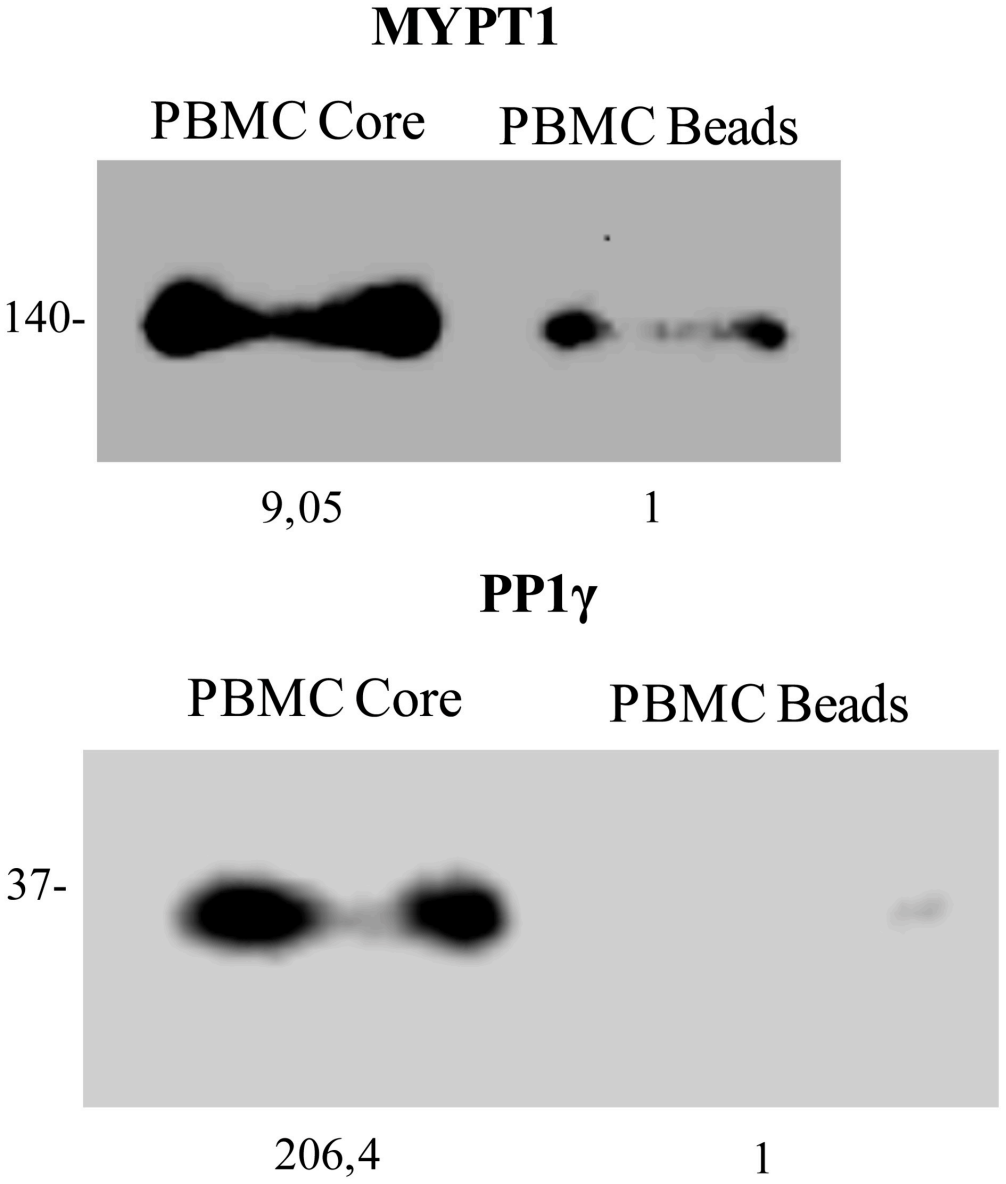
HCV Core protein interacts with PP1γ and MYPT1 in PBMC blasts. Postnuclear lysates from Human PBMCs stimulated with PHA and cultured during 5 days with IL-2, were subjected to HCV Core protein pull down. Proteins pulled down by HCV Core coated magnetic beads (PBMC Core band) or by uncoated magnetic beads (PBMC beads band) were analyzed by Western blot with anti-human MYPT1 **(A)** and anti-human PP1γ **(B)**. Molecular mass, in kDa, is indicated on the side and band quantitation at the bottom for each Western blot.

Serine/threonine-protein phosphatase was selected due to its known localization (it has a dynamic and predominantly nucleolar distribution) and to its function (it has been implicated in the regulation of several biological pathways previously described in T cells transduced with HCV Core protein) (Aggen et al., 2000; Ceulemans and Bollen, 2004; Nie et al., 2013). It was also selected based on the fact that both its catalytic subunit (PP1γ) and its regulatory subunit 12 A (MYPT1), were identified by Mass spectrometry, showing a despairing Mascot Score of 37.6 and 336.5 respectively. As shown in Figure 4, Western blot analysis of (PP1γ) and (MYPT1), confirmed the presence of both proteins in PBMCs lysates obtained from the pull down of HCV Core protein coated magnetic beads (Figure 4).

### Two nucleolar localization sequences (NolSs) of HCV core protein were determined using a bioinformatics tool

**Figure 5:**
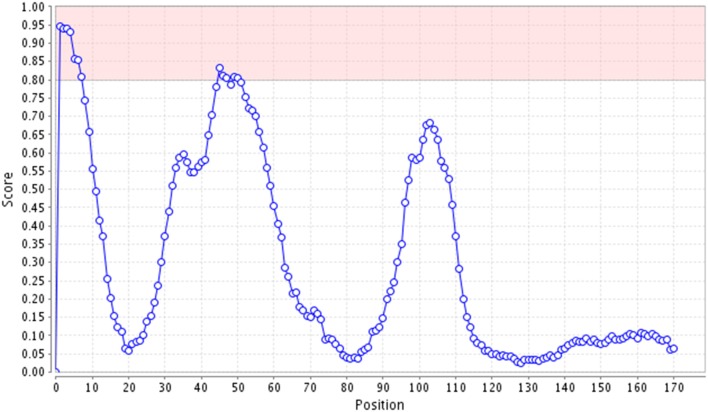
Predicted Nucleolar localization sequences in HCV core protein. Graph obtained from the Nucleolar Localization Sequence detector web server (NoD), displaying NoLS prediction score for each residue of HCV Core protein. Pink shaded regions represent the range of scores within which a 20-residues segment is predicted to be a NoLS. Thus, pink shaded regions represent the NoLS candidate segment, which highlights scores above 8.0.

In order to identify potential nucleolar localization sequences in HCV core protein, we used the NoD web server (http://www.compbio.dundee.ac.uk/www-nod/), a program that predicts the presence of NoLSs in eukaryotic and viral proteins, based on a database of 46 statistically analyzed human nucleolar localization sequences (Scott et al., 2011). Two NoLSs were identified in HCV core protein (Figure 5) including sequence: STNPKPQRKTKRNTNRRPQDVKFPGG (between positions 1 and 26) and VRATRKTSERSQPRGRRQPIPKARQ (between positions 45 and 69).

NoLS sequences were highlighted in the context of the full-length protein in order to visualize its position: **STNPKPQRKTKRNTNRRPQDVKFPGG**GQIVGGVYLLPRRGPRLG**VRATRKTSERSQPRGRRQPIPKARQ**PEGRAWAQPGYPWPLYGNEGLGWAGWLLSPRGSRPSWGPTDPRRRSRNLGKVIDTLTCGFADLMGYIPLVGAPLGGAARALAHGVRVLEDGVNYATGNLPGCSFSIFLLALLSCLTIPASA.

## Discussion

Several biological and immunological consequences of HCV core intracellular expression in CD4^+^ T cells have previously been described by us and others, (Bergqvist and Rice, 2001; Bergqvist et al., 2003; Dominguez-Villar et al., 2007, 2012a; Doumba et al., 2013; Fernandez-Ponce et al., 2014). These findings suggest an important role for the intracellular presence of HCV core in HCV pathogenesis and chronification. In this study, we have analyzed the ultrastructural localization of HCV core protein in CD4^+^ T cells.

According to studies using cell lines unrelated to the immune system, HCV core protein has been shown to localize in the endoplasmic reticulum, mitochondrial outer membrane and nucleus of Human embryonic kidney 293T cells (Suzuki et al., 2005); associated to lipid droplets in CHO, HepG2 and Huh7 cells lines (Barba et al., 1997; Boulant et al., 2006; Qiang and Jhaveri, 2012), in the cytoplasm, endoplasmic reticulum, in the proximity of the nuclear membrane, in the nucleus and nucleoli of hepatocytes isolated from chronically HCV infected patients (Falcon et al., 2003); and in the cytoplasm and nucleus of non-parenchymal liver cells such as, lymphocyte-like cells, Kupffer-like cells, polymorphonuclear-like cells, pit, endothelial, stellate, and fibroblast-like cells isolated from livers of chronically HCV infected patients (Falcon et al., 2005).

In the present work, we found that in CD4^+^ T lymphocytes, HCV core protein mostly localizes in the nucleus and specifically in the nucleolus where it is greatly enriched and mainly organized in clusters (Figure 1). Regarding these findings, Nuclear Localization Signals (NLSs) described previously in HCV core protein, could be responsible for nuclear localization (Chang et al., 1994; Suzuki et al., 1995, 2005), while HCV core protein traffic and residence in the nucleolus, could be explained by the presence of two Nucleolar Localization Sequences (NoLSs), identified using the bioinformatic Nucleolar localization sequence detector web server for eukaryotic and viral proteins (Scott et al., 2011). NolSs are showed in Figure 4. The statistical variation in Core protein nucleolar localization shown by HCV-Core expressing cells, could be due to differences in cellular cycle stage, as it has been described for other nucleolar resident proteins (Chen and Huang, 2001; Stoldt et al., 2007; Pirlot et al., 2016). Presence of NoLSs in cellular or viral proteins is a key factor in their dynamic traffic and residence within the nucleolus. Presumably, NoLSs interact with nucleolar proteins and/or RNA to mediate nucleolar targeting and retention. Thus, in chimeric viral proteins with mutated NoLSs, trafficking to the nucleolus is abrogated, and heterologous NoLSs insertion restores their trafficking pattern (Boyne and Whitehouse, 2006; Emmott et al., 2008).

Interestingly, the function of HCV core protein inside the virus, can partly explain its subcellular localization as HCV core protein mainly interacts with HCV genomic RNA, multimerizing around it and forming the capsid shell. While multimerizing inside the virus, we have not seen any multimerization in our experiments, which is in agreement with several studies showing that mammalian cell lines fail to produce capsid assembly (Bukh et al., 2002; Pietschmann et al., 2002; Polyak et al., 2006; Rouille et al., 2006; Hourioux et al., 2007), due to the lack of host cells factors that are essential for HCV Core multimerization and assembly, or to the presence of inhibitory factors that induce the majority of HCV-core to be targeted away from the ER (Hope and McLauchlan, 2000; McLauchlan et al., 2002; Polyak et al., 2006). Thus, un-multimerized HCV Core protein traffics to alternate subcellular compartments. HCV core has been shown to bind RNAs in addition to HCV genomic RNA (Kunkel et al., 2001; Cristofari et al., 2004), including ribosomal RNA (Santolini et al., 1994) and tRNA (Kunkel et al., 2001). Since the nucleolus contains several 100 copies of rRNA genes and it could be a recruiting site for tRNAs (Carmo-Fonseca et al., 2000) it is likely that the nucleolar RNA constitutes another important molecular target structure for HCV core protein.

In agreement with our findings, several DNA viruses, retroviruses and RNA viruses, as well as viral proteins, have been described to traffic to the nucleolus and be associated with nucleolar proteins (Hiscox et al., 2001, 2010; Wurm et al., 2001; Chen et al., 2002; Dove et al., 2006; Michienzi et al., 2006; Cawood et al., 2007; Hiscox, 2007; Emmott et al., 2010; Lam et al., 2010; Jarboui et al., 2012).

The nucleolus is a dynamic nuclear organelle whose proteome is continuously changing. It seems to be a temporary storage or a sequestration site for a multiplicity of proteins (Emmott and Hiscox, 2009; Hiscox et al., 2010). Functionally, there is extensive evidence that the nucleolus is implicated in ribosome biogenesis (Stoykova et al., 1985; Warner, 1990; Scheer et al., 1993), cell cycle regulation, cell growth, senescence, stress response signaling (Andersen et al., 2005; Emmott and Hiscox, 2009; Tsai and Pederson, 2014; Lam and Trinkle-Mulcahy, 2015) and the pathogenesis of several diseases such as cancer (James et al., 2014; Orsolic et al., 2015; Yang et al., 2015), cardiovascular disease (Hariharan and Sussman, 2014) and neurodegenerative disorders (Payao et al., 1994; Lu et al., 1998; Rieker et al., 2011; Tsoi and Chan, 2013; Lee et al., 2014a; Parlato and Liss, 2014; Hernandez-Ortega et al., 2015).

Viral protein trafficking and localization into the nucleolus has shown implications in both viral life cycle and in host cell physiology and it has been narrowly related with the loss of essential nucleolar functions (Hiscox, 2002). In addition, it has been shown that accumulation of viral proteins in the nucleolus can cause volume exclusion and crowding effects, disrupting the nucleolar architecture (Hancock, 2004; Hiscox, 2007). Virus infection and some viral proteins from Poliovirus, Avian Infectious Bronchitis Virus (IBV), Coronavirus and Human Immunodeficiency Virus-1 (HIV-1) induce disruption of the nucleolar architecture and changes on the subcellular distribution of nucleolar proteins or proteins that traffic to the nucleolus, such as nucleolin, p53, B23.1 (Waggoner and Sarnow, 1998; Hiscox, 2002; Dove et al., 2006). These findings are closely related to the presence of perturbations in cell cycle, cytokinesis and apoptosis in the host cells (Miyazaki et al., 1996; Chen et al., 2002; Galati et al., 2003; Hiscox, 2007).

Semliki Forest Virus nucleocapsid migrates to the nucleolus (Jakob, 1994) and Porcine Reproductive and Respiratory Syndrome Virus nucleocapsid specifically interacts with the small nucleolar RNA (snoRNA)-associated protein, fibrillarin in virus infected cells (Rowland et al., 1999; Yoo et al., 2003), while Hepatitis B Virus (HBV) core protein usually co-localizes with the nucleolar proteins, nucleolin and B23 (Ning and Shih, 2004). In addition, it has been shown that Coronavirus nucleocapsid protein localization in the nucleolus of infected cells and its association to the nucleolar protein B23.1 is related to cell cycle stage and could be involved in cell cycle delay or arrest to promote virus replication (Hiscox et al., 2001; Wurm et al., 2001; Cawood et al., 2007).

Thus, trafficking to the nucleolus and nucleolar residency time of HCV core protein in CD4^+^ T cells could be narrowly related with previous findings from us and others showing that the intracellular presence of HCV core in CD4^+^ T cells induces decreasing cell proliferation, delay in cell cycle progression and a differential expression pattern of genes with relevant function, including anergy-associated genes, genes involved in cytoskeleton reorganization, vesicle trafficking, endocytosis, cytokines production, cell death, transcription, and translation (Dominguez-Villar et al., 2007).

In agreement with the described localization, we found HCV core protein association with several nuclear, nucleolar proteins or proteins described to traffic to the nucleolus, which showed mainly binding connections (Figure 2). The wide range of proteins associated with HCV core, correlate with the findings obtained by Dolan et al. who identified two computationally predicted molecular recognition features within the N-terminal intrinsically disordered region (IDR) in the HCV core sequence. The identified molecular recognition features, mediate HCV core protein binding to HCV RNA and to multiple host proteins, suggesting that HCV core protein exhibits Hub protein properties (Dolan et al., 2015).

Interestingly, pathway and network-based analysis of proteins identified to be associated with HCV Core protein, indicate that a wide range of these proteins are involved in several biological signaling pathways, such as, RNA processing and splicing (Figure 3A), cell cycle progression, cell proliferation, apoptosis (Figure 3B) and infection and replication of RNA viruses, including HCV (Figure 3C). With the integrated analysis of the present data, we confer a better description of the HCV Core -human T lymphocyte relationship.

Concerning ribosomal biogenesis, cellular proliferation, cell cycle and apoptosis; several large (RPL) and small (RPS) ribosomal proteins, and proteins involved in RNA processing and splicing as DEAD-box RNA helicases, precipitated with HCV core (Figures 3A,B) (Rocak and Linder, 2004; Xu et al., 2016). Interactions between viral and ribosomal proteins, splicing factors and DEAD-box RNA helicases including DDX5 and DDX17, have been previously described and suggested as a key mechanism for viral replication and production as well as for life cycle progression and survival (Bortz et al., 2011; Naji et al., 2012; Yasuda-Inoue et al., 2013; Cervantes-Salazar et al., 2015; Klymenko et al., 2016; Li et al., 2016). Implication of other identified proteins, such as Protein Red (IK) and Filamin A (FLNA) (Figure 3B) in proliferation and cell cycle progression, have also been widely demonstrated (Lee et al., 2014b; Sun et al., 2014). Thus, associations of HCV core protein in T cells could alter the function of the associated proteins, explaining some of the effects shown for HCV-core expression, including cell cycle delay and inhibition of cell proliferation (Dominguez-Villar et al., 2007, 2012a; Fernandez-Ponce et al., 2014).

In addition, we found that many host proteins associated with HCV core,¯ are involved in replication and infection of RNA viruses including HCV (Figure 3C). Proteins as DExD/H-box helicases have been extensively studied in virus infection and have been described as proteins hijacked by viruses for their benefit. Specifically, DHX9 is involved in virus replication, innate immunity response to viral dsRNA and participation in the expression of IFN-stimulated genes (Fullam and Schroder, 2013). RNA Binding Proteins (RBPs) such as the host Poly(rC)-Binding Protein (PCBP) and different Heterogeneous Nuclear Ribonucleoproteins (HNRPs), are also interesting as they stabilize viral RNAs, co-localize with the viral replicase complex and facilitate viral RNA template selection (Li and Nagy, 2011). In addition, Interleukin enhancer-binding factor 3 (ILF3) has been shown to be recruited to HCV replication complexes (Li et al., 2014) and in other infections by RNA viruses, ILF3 interaction with viral proteins has been described to affect virus replication among other virus life cycle stages (Patino et al., 2015).

Furthermore, the subcellular localization of HCV core and its association with nuclear and nucleolar proteins found in the present study, can aid in explaining the CD4^+^ T cell regulatory/exhausted phenotype described by us and others, in CD4^+^ T cells expressing HCV core protein (Dominguez-Villar et al., 2012a; Doumba et al., 2013; Fernandez-Ponce et al., 2014). Some such associations are with serine/threonine-protein phosphatase catalytic subunit (PP1γ), protein phosphatase 1 regulatory subunit 12A (MYPT1) (Nie et al., 2013), interleukin enhancer-binding factor 3 (ILF3) (Shi et al., 2007a,b), complement component C1Q binding protein (C1QBP) (Kittlesen et al., 2000) and runt-related transcription factor 1 (RUNX1) (Klunker et al., 2009).

In conclusion, analysis of the association of HCV core with host proteins in CD4^+^ T cells and the study of its ultrastructural localization, open an extensive field of study poised to understand the mechanisms underlying functional findings previously described in CD4^+^ T cells expressing HCV core protein, that have demonstrated to be relevant for HCV immune system evasion and thus HCV chronification.

## Author contributions

Conception, design and or interpretation of the work: FG-C, EA, and RL. WB (for localization studies). CB and MD-R (for association studies). Performed association studies: CF-P, MD-R, and AS-S. Performed localization studies: CF-P, JM-M, and MA-E. Run statistical analyses: RL and CF-P. Wrote the paper: FG-C, CF-P, EA, RL, and MD-R. Performed confirmation co-IP experimentos: IN-S and CF-P. All authors participated in critical revision and subsequently approved the manuscript. All authors agree to be accountable for all aspects of the work in ensuring that questions related to the accuracy or integrity of any part of the work are appropriately investigated and resolved.

### Conflict of interest statement

The authors declare that the research was conducted in the absence of any commercial or financial relationships that could be construed as a potential conflict of interest.
